# Do Additional Clinical Risk Factors Improve the Performance of Fracture Risk Assessment Tool (FRAX) Among Postmenopausal Women? Findings From the Women's Health Initiative Observational Study and Clinical Trials

**DOI:** 10.1002/jbm4.10239

**Published:** 2019-11-30

**Authors:** Carolyn J Crandall, Joseph Larson, Jane A Cauley, John T Schousboe, Andrea Z LaCroix, John A Robbins, Nelson B Watts, Kristine E Ensrud

**Affiliations:** ^1^ Division of General Internal Medicine and Health Services Research David Geffen School of Medicine at University of California Los Angeles CA USA; ^2^ Fred Hutchinson Cancer Research Center Seattle WA USA; ^3^ Department of Epidemiology, Department of Epidemiology Graduate School of Public Health, University of Pittsburgh Pittsburgh PA USA; ^4^ HealthPartners Institute, Park Nicollet Clinic, and University of Minnesota Minneapolis MN USA; ^5^ Department of Family and Public Health University of California, San Diego La Jolla CA USA; ^6^ Department of Medicine UC Davis Medical Center Sacramento CA USA; ^7^ Mercy Health Osteoporosis and Bone Health Services Cincinnati OH USA; ^8^ Division of Epidemiology & Community Health University of Minnesota Medical School Minneapolis MN USA

**Keywords:** DIABETES, FALLS, FRACTURE, FUNCTIONAL STATUS, HOT FLASHES, OSTEOPOROSIS, VASOMOTOR SYMPTOMS

## Abstract

The ability of the fracture risk assessment tool (FRAX) to discriminate between women who do and do not experience major osteoporotic fractures (MOFs) is suboptimal. Adding common clinical risk factors may improve discrimination. We used data from the Women**'**s Health Initiative, a prospective study of women aged 50 to 79 years at baseline (*n* = 99,413; *n* = 5722 in BMD subset) enrolled at 40 US clinical centers. The primary outcome was incident MOFs assessed annually during 10 years**'** follow‐up. For prediction of incident MOF, we examined the area under the receiver operatic characteristic curve (AUC) and net reclassification index (NRI) of the FRAX model alone and FRAX plus additional risk factors (singly or together: type 2 diabetes mellitus, frequent falls [≥2 falls in the past year], vasomotor symptoms, self‐reported physical function score [RAND 36‐item Health Survey subscale), and lumbar spine BMD). For NRI calculations, high risk was defined as predicted MOF risk ≥20%. We also assessed calibration as observed MOF events/expected MOF events. The AUC value for FRAX without BMD information was 0.65 (95% CI, 0.65 to 0.66). Compared with the FRAX model (without BMD), the AUC value was not improved by the addition of vasomotor symptoms, diabetes, or frequent falls, but was minimally increased by adding physical function score (AUC 0.66, 95% CI, 0.66 to 0.67). FRAX was well‐calibrated for MOF prediction. The NRI of FRAX + additional variables versus FRAX alone was 5.7% (*p* < 0.001) among MOF cases and −1.7% among noncases (*p* > 0.99). Additional variables (diabetes, frequent falls, vasomotor symptoms, physical function score, or lumbar spine BMD) did not yield meaningful improvements in NRI or discrimination of FRAX for MOFs. Future studies should assess whether tools other than FRAX provide superior discrimination for prediction of MOFs. © 2019 The Authors. *JBMR Plus* published by Wiley Periodicals, Inc. on behalf of American Society for Bone and Mineral Research.

## Introduction

The fracture risk assessment tool, FRAX, is a web‐based clinical tool that uses individual clinical risk factors to predict the 10‐year risk of hip fracture and the 10‐year risk of major osteoporotic fracture (MOF; clinical spine, forearm, hip, or shoulder fracture).^1^ The FRAX prediction tool can be used either with or without femoral neck BMD information. Several US clinical guidelines about osteoporosis screening and treatment recommend the use of the FRAX tool in clinical decision‐making. For example, the United States Preventive Services Task Force recommends BMD testing for postmenopausal women aged 50 to 64 years who have a 10‐year FRAX‐predicted risk of MOF ≥8.4% (calculated using FRAX without BMD information).^2^ However, the performance of the FRAX (with or without BMD) in discriminating between women who will or will not experience a subsequent MOF is suboptimal (area under the receiver operating characteristic curve [AUC] value <0.65).^3^ Among younger postmenopausal women aged 50 to 64 years, the ability of FRAX to discriminate between women who will or will not experience MOF is no better than chance alone (AUC approximately 0.56).^4^, ^5^ Similar findings have been reported for women aged ≥65 years.^6^ Therefore, there is room for improvement in fracture risk discrimination by FRAX.

Because data were not consistently available in its development cohort, FRAX does not include several known fracture risk factors, such as type 2 diabetes^7^, ^8^, ^9^ and falls.^9^, ^10^ Also, FRAX is not validated for use with lumbar spine BMD.^1^ If lumbar spine BMD is lower than femoral neck BMD, FRAX will underestimate major osteoporotic fracture risk.^11^, ^12^ In the National Health and Nutrition Examination Survey 2005 to 2008, roughly one‐third of US women aged ≥50 years differed in skeletal status at the lumbar spine and hip, with most being normal at one site and having *T*‐score ≤ −1.0 at the other site.^13^ Women who are osteoporotic only at the spine may have not have been identified from hip BMD measurement alone, yet they may have high enough fracture risk to warrant consideration of treatment.^14^ Finally, a previous report from the Women**'**s Health Initiative (WHI) study reported that women with vasomotor symptoms (hot flashes and/or night sweats) have lower BMD and higher fracture risk than women without vasomotor symptoms.^15^, ^16^

Using data from the WHI, we examined measures of discrimination, calibration, and net risk reclassification to evaluate whether the addition of selected risk factors (frequent falls, type 2 diabetes mellitus, vasomotor symptoms, impaired physical function, and lumbar spine BMD) to FRAX improved model performance for prediction of risk of subsequent MOF.

## Subjects and Methods

### The Women**'**s Health Initiative study design

The WHI study enrolled 161,808 postmenopausal women aged 50 to 79 years at baseline at 40 US clinical centers. The study design of WHI has been previously described.^17^, ^18^, ^19^ Participants were free from serious medical conditions. The WHI consisted of an observational study (WHI‐OS) and three clinical trials (WHI‐CTs) that evaluated a low‐fat eating pattern, menopausal hormone therapy, and calcium + vitamin D supplementation.

For the WHI Bone Density substudy, at the time of enrollment, all WHI‐OS and WHI‐CT participants at 3 of the 40 clinical centers underwent hip and lumbar spine BMD testing.

**Figure 1:**
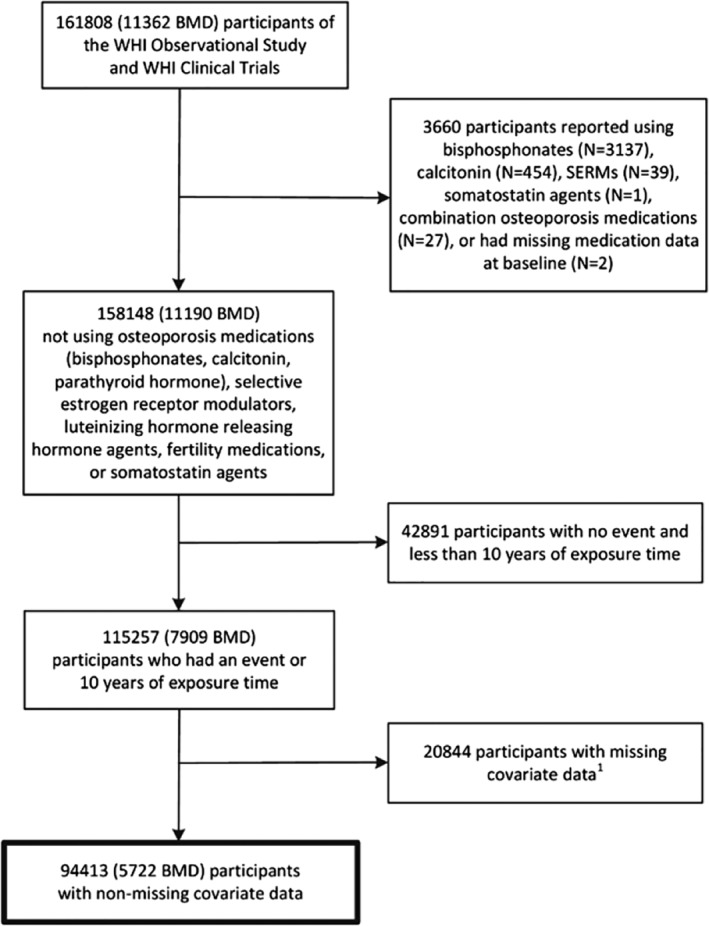
Flow diagram of the analytic cohort. Missing covariate data: history of treated diabetes, falls in the past year, vasomotor symptoms, physical function, history of fracture ≥55, and BMI. WHI = Women**'**s Health Initiative; BMI = body mass index.

Of the 161,808 participants of the WHI‐OS and WHI CTs, we excluded data from participants who reported using osteoporosis medication (bisphosphonates, selective estrogen receptor modulators, calcitonin, parathyroid hormone/teriparatide, or denosumab) at baseline (*n* = 3660), participants who provided less than 10 years of follow‐up time without experiencing a MOF (*n* = 42,891), and participants for whom information regarding osteoporosis risk factors was missing (*n* = 20,844), yielding an analytic sample size of 99,413 women (Fig. 1). For the analyses focused on FRAX models with BMD information, we included data from all participants of the WHI Bone Density substudy (*N* = 5722). Human subjects**'** review committees at each participating institution reviewed and approved the study. Each participant provided written informed consent.

### Fracture ascertainment

Information regarding fractures was self‐reported on questionnaires administered at baseline and annually. Participants were asked, “Since the date on the front of this form, has a doctor told you for the time that you have a new broken, crushed, or fractured bone?” Response choices included hip, upper leg, pelvis, knee (patella), lower leg or ankle, foot (not toe), tailbone (coccyx), spine or back (vertebra), lower arm or wrist, hand (not finger), elbow, upper arm, or shoulder.

Hip fractures were confirmed using medical records, but other types of fractures were self‐reported.

### Bone mineral density measurement

For the WHI Bone Density substudy, at the time of enrollment, all WHI‐OS and WHI‐CT participants at 3 of the 40 clinical centers (Tucson/Phoenix, AZ, USA; Pittsburgh, PA, USA; and Birmingham, AL, USA) underwent hip and anteroposterior lumbar spine BMD testing using DXA (Hologic QDR2000 or QDR4500; Hologic, Inc., Waltham, MA, USA).^20^, ^21^ Standardized protocols were used for participant positioning and analysis. Cross‐calibration phantoms, further evaluation of scans with specific problems, and review of a random sample of scans were included in the quality assurance methods.^20^, ^21^, ^22^

### Data collection regarding fracture risk factors

Information regarding age, race/ethnicity, socioeconomic status, medical history (including fracture prior to baseline), reproductive history, family medical history, frequency of falls, medication use, alcohol intake, smoking, vasomotor symptoms, general health status (excellent, very good, fair, or poor), physical activity,^23^ dietary and alcohol intake,^24^ and dietary supplement use was collected on baseline questionnaires. The RAND 36‐item Health Survey (SF‐36) physical function subscale was used to assess self‐reported physical function.^25^ FRAX‐predicted 10‐year risk of a MOF was calculated at baseline using FRAX version 3.0.^26^

Height and body weight were measured at baseline. BMI was calculated as body weight in kg divided by the square of the height in cm.

### Statistical analysis

To determine model discrimination, we calculated the area under the receiver operating characteristic (AUC) curve values for models based on FRAX alone and those based on FRAX + additional risk factors for distinguishing between women who did and did not experience incident MOF during the 10‐year study follow‐up period. Risk factors were added one at a time, as well as simultaneously to the FRAX model. The candidate additional risk factors, selected a priori based on previously published studies, were diabetes mellitus,^27^ ≥2 falls in the prior year,^28^ vasomotor symptoms,^16^ poor physical function score (continuous),^29^ and lumbar spine BMD (in the BMD subset).^14^ We also compared AUC values for the FRAX model with those of simpler models: age alone and age + BMI. We then repeated the same procedure with the secondary outcome of incident hip fracture.

The primary analyses included all participants who provided 10 years of follow‐up information regarding incident fractures. However, in sensitivity analyses, we recalculated AUC values with inclusion of participants with less than 10 years of follow‐up regardless of fracture status (*N* = 35,026 additional participants, 9599 of whom died within 10 years of study enrollment). In additional sensitivity analyses, we repeated the primary analysis among women aged 65 and older and among nonusers of menopausal hormone therapy (excluding women who reported hormone therapy use at baseline or who were assigned to hormone therapy in the WHI hormone therapy clinical trials).

Net reclassification indices (NRIs) comparing the nested models (FRAX versus FRAX + additional risk factors) were calculated separately for cases (women with an incident MOF event during the 10‐year follow‐up period) and noncases (women without an incident MOF).^30^, ^31^ We designated “high risk” as predicted MOF risk ≥20% and “low risk” as predicted MOF risk <20%. The NRI components express the net percentage of components with or without events that are correctly reclassified.^32^ Negative percentages for the components signify a net worsening in risk classification. The event NRI can be interpreted as the improvement in sensitivity, whereas the nonevent NRI is the improvement in specificity. We also performed NRI calculations for hip fracture risk, where high risk was predicted as predicted hip fracture risk ≥3% and low risk was predicted hip fracture risk <3%.

We assessed the calibration (actual observed versus predicted risk of MOF) of the FRAX fracture risk prediction tool and the model that included FRAX + the additional risk factors by dividing the population into deciles defined by the level of predicted fracture risk. We created a figure in which each data point represents proportions of individuals observed and predicted to have the outcome of interest (MOF) within a decile of risk.^30^ The lowest decile of risk represents the 10% of women with the lowest predicted probability of MOF (data point at the far lower left) and the highest decile of risk represents the 10% of individuals with the highest predicted probability of MOF (data point at the far higher right). We used the same method to perform calibration calculations for observed versus predicted risk of hip fracture.

## Results

The characteristics of the overall analytic sample (*n* = 94,413) and the BMD subset (*n* = 5722) are displayed in Table 1. Out of 17,435 participants with at least one MOF event, there were 1978 hip fractures, 5534 lower arm/wrist fractures, 2445 upper arm fractures, and 2877 clinical vertebral fractures. The mean age (SD) of participants was 63.0 (7.0) years; 29% of participants had BMI ≥30 kg/m^2^; 87% of participants were white; 10.4% (*n* = 7884) of participants were black or Hispanic.

**Table 1:** Baseline Characteristics of Both the Overall and BMD Subset

	Overall sample (*n* = 94,413)		BMD subset (*n* = 5722)	
Baseline characteristic	*n*	%^a^	*n*	%
Age, years, mean (SD)	63.0	(7.0)	63.0	(7.1)
Race/Ethnicity				
Black	6303	6.7	672	11.7
Hispanic	2581	2.7	253	4.4
White	81,947	86.8	4695	82.1
Other / unknown	3582	3.8	102	1.8
BMI, kg/m^2^, mean (SD)	27.8	(5.8)	28.0	(5.8)
<25	34,507	36.5	1958	34.2
25–<30	32,972	34.9	2011	35.1
≥30	26,934	28.5	1753	30.3
Lumbar spine BMD, g/cm^2^, mean (SD)	1.0	(0.2)	1.0	(0.2)
Physical function score, mean (SD)	83.0	(18.5)	80.9	(19.6)
Smoking				
Never	48,158	51.0	3130	54.7
Past	39,767	42.1	2134	37.3
Current	5604	5.9	392	6.9
Alcohol use				
Never	8993	9.5	843	14.7
Past	15,781	16.7	1144	20.0
Current (<1 drink/month)	69,223	73.3	3694	64.6
Hormone therapy use^b^	47,364	50.2	2675	46.7
Daily glucocorticoid use^c^	315	0.3	21	0.4
Falls in the past year				
0	64,358	68.2	3966	69.3
1	18,681	19.8	1096	19.2
2	7703	8.2	430	7.5
≥ 3	3671	3.9	230	4.0
History of fracture ≥ age 55				
Yes	8660	9.2	554	9.7
No	73,506	77.9	4373	76.4
Not applicable (<55 years old)	12,247	13.7	795	13.9
Parental history of hip fracture	12,890	13.7	805	14.1
Hysterectomy	38,471	40.7	2791	48.8
Early menopause (≤45 years old)	19,412	20.6	1369	23.9
Current vasomotor symptoms	21,529	22.8	1292	22.6
History of rheumatoid arthritis	4190	4.4	295	5.2
History of malabsorption^d^	283	0.3	15	0.3
History of liver disease	2053	2.2	127	2.2
History of emphysema	2920	3.1	185	3.2

a Percentages may not add up to 100% because of missing data.

b Hormone use incorporates both a participant**'**s self‐report status at baseline as well as her intervention assignment in the Women**'**s Health Initiative Hormone Therapy trial. Women assigned to active hormone therapy intervention were categorized as “Yes” for hormone therapy use, while women assigned to placebo were categorized as “No.” Women not in the Hormone Therapy trial were assigned their baseline self‐report hormone use.

c Glucocorticoid use defined as ≥3 months of daily oral use of ≥5 mg prednisone or equivalent.

d Self‐report of special diet prescribed for malabsorption, celiac sprue, ulcerative colitis, or Crohn**'**s disease.

### FRAX without BMD models: Discrimination for MOF and for hip fractures

In the overall analytic sample, FRAX (without BMD information) had suboptimal ability to distinguish between women who did and did not experience a MOF (AUC 0.65; 95% CI, 0.65 to 0.66). The AUC value for FRAX was essentially identical to that for age alone (AUC 0.65; 95% CI, 0.64 to 0.65), and age + BMI (AUC 0.65; 95% CI, 0.64 to 0.65; Table 2). Compared with the AUC value for FRAX (without BMD) alone in predicting a MOF, AUC values were not improved by the addition of vasomotor symptoms, diabetes, or frequent falls (≥2 falls in the past year), individually or simultaneously, to the FRAX model. The AUC value of the model containing FRAX (without BMD) was minimally increased by the addition of physical function score (AUC 0.66; 95% CI, 0.66 to 0.67). The same pattern was apparent for hip fractures; AUC values for FRAX were not improved by the addition of vasomotor symptoms, diabetes, or frequent falls either individually or simultaneously, to the FRAX model.

**Table 2:** Area Under the Receiver Operating Characteristic Curve Values for Fracture Risk Assessment Tool (FRAX) Alone and FRAX with Additional Clinical Characteristics on Predicted 10‐Year Risk of Fracture

	Hip fracture		Major osteoporotic fracture	
Model^a^	*n*	AUC (95% CI)	*n*	AUC (95% CI)
All participants				
Age	92,075	77.1 (76.0–78.2)	94,413	64.7 (64.1–65.2)
Age + BMI	92,075	77.4 (76.3–78.4)	94,413	64.6 (64.1–65.2)
Age + history of fracture (any site)	92,075	77.1 (76.1–78.2)	94,413	65.2 (64.6–65.7)
FRAX alone (all participants)	92,075	76.2 (75.1–77.3)	94,413	65.0 (64.5–65.6)
FRAX alone (white participants)	79,909	75.4 (74.3–76.6)	81,947	64.2 (63.6–64.8)
FRAX alone (black participants)	6194	81.2 (74.5–88.0)	6903	60.6 (57.5–63.7)
FRAX + treated diabetes	92,075	76.5 (75.4–77.5)	94,413	65.3 (64.8–65.9)
FRAX + ≥2 falls in the past year	92,075	75.4 (74.3–76.5)	94,413	65.3 (64.8–65.9)
FRAX + vasomotor symptoms	92,075	74.8 (73.6–76.0)	94,413	65.0 (64.4–65.5)
FRAX + physical function	92,075	75.9 (74.9–77.0)	94,413	66.2 (65.6–66.7)
FRAX + all additional factors	92,075	76.2 (75.1–77.3)	94,413	66.6 (66.0–67.1)
BMD subset				
Age	5541	76.1 (72.1–80.2)	5722	66.2 (64.1–68.3)
Age + BMI	5541	76.3 (72.2–80.4)	5722	66.2 (64.1–68.3)
Age + history of fracture (any site)	5541	76.4 (72.5–80.4)	5722	67.4 (65.3–69.5)
FRAX alone (all participants)	5541	77.6 (73.7–81.5)	5722	69.8 (67.8–71.8)
FRAX alone (white participants)	4537	77.0 (72.8–81.1)	4695	68.3 (66.1–70.5)
FRAX alone (black participants)	663	84.5 (69.2–99.9)	672	65.6 (55.6–75.6)
FRAX + treated diabetes	5541	77.2 (73.1–81.2)	5722	70.2 (68.2–72.1)
FRAX + ≥2 falls in the past year	5541	76.6 (72.6–80.7)	5722	70.0 (68.0–72.0)
FRAX + vasomotor symptoms	5541	76.4 (72.1–80.7)	5722	69.8 (67.8–71.8)
FRAX + physical function	5541	77.3 (73.4–81.2)	5722	70.7 (68.7–72.7)
FRAX + lumbar spine BMD	5541	75.9 (71.7–80.1)	5722	70.0 (68.0–71.9)
FRAX + all additional factors	5541	77.7 (73.4–81.9)	5722	71.4 (69.4–73.4)

a All models are adjusted for intervention assignment in the WHI Hormone (Active, Placebo, not Randomized) and Calcium Vitamin D (Active, Placebo, Not Randomized) trials.

Discrimination for a MOF by FRAX without BMD information was higher among white participants (AUC 0.64; 95% CI, 0.64 to 0.65) than among black participants (AUC 0.61; 95% CI, 0.58 to 0.64). In contrast to the pattern for a MOF, the FRAX AUC values for hip fracture were higher among black participants (AUC 81.2; 95% CI, 74.5 to 88.0) than among white participants (AUC 75.4; 95% CI, 74.3 to 76.6). AUC values were higher for hip fractures than for a MOF, generally ranging from 75 to 77.

### FRAX with BMD information: Discrimination for MOF and for hip fractures

**Figure 2:**
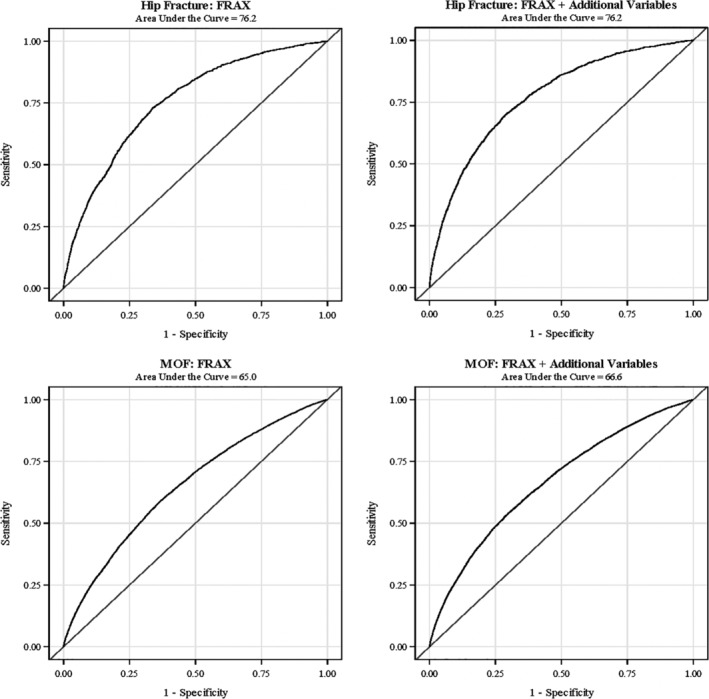
Receiver operatic characteristic curves for the prediction of 10‐year fracture incidence with FRAX alone, and fracture risk assessment tool (FRAX) with additional clinical characteristics: treated diabetes, ≥ two falls in the past year, vasomotor symptoms, physical function score. All models are adjusted by the Women**'**s Health Initiative hormone and calcium plus vitamin D intervention status (active, placebo, not randomized).

Compared with age alone (AUC 0.66; 95% CI, 0.64 to 0.68) and age + BMI (AUC 0.66; 95% CI, 0.64 to 0.68), discrimination for a MOF was slightly higher (although still suboptimal) for FRAX with BMD information (AUC 0.70; 95% CI, 0.68 to 0.72). Discrimination for a MOF by FRAX with BMD information was higher among white participants (AUC 0.69; 95% CI, 0.66 to 0.71) than among black participants (AUC 0.66; 95% CI, 0.56 to 0.76). The AUC value for white participants was statistically significantly higher than the AUC value for black participants (*p*‐value = 0.03 for two‐sided chi‐square test with a null hypothesis that AUC white = AUC black). In the overall cohort, the addition of diabetes, frequent falls, vasomotor symptoms, physical function, and lumbar spine BMD, either individually or together, to FRAX did not notably increase AUC values for MOF as compared with values for the FRAX model with BMD. AUC curves for the prediction of incident fractures during 10‐year follow‐up are displayed in Fig. 2.

As was the case for FRAX without BMD information, AUC values for FRAX with BMD information were higher for hip fractures than for MOF, generally ranging from 76 to 78, and the addition of diabetes, falls, vasomotor symptoms, and physical function (either individually or simultaneously) did not improve AUC values for the prediction of hip fracture. Hip fracture AUC values were higher among black participants (AUC 84.5; 95% CI, 69.2 to 99.9) than among white participants (AUC 77.0; 95% CI, 72.8 to 81.1).

### Reclassification of risk by FRAX and FRAX plus additional variables

Among cases, 959 of 11,710 participants (8.1%) who experienced a MOF were correctly reclassified up (from low to high risk) with the FRAX + additional factors model compared with the FRAX model alone (Table 3). Conversely, 291 of 11,710 participants (2.1%) who experienced a MOF were incorrectly reclassified down (from high to low risk) with the FRAX + additional factors model. The NRI value for cases (women with a MOF) was 5.7%, that is a 5.7% improvement in reclassification among cases. Among noncases, 2747 participants who did not experience a MOF were incorrectly reclassified up (from low to high risk) with the FRAX + additional factors model, and 1332 participants who did not experience a MOF were correctly reclassified down (from high to low risk) with the FRAX + additional factors model. The NRI value for noncases was −1.7%, that is a 1.7% worsening in reclassification among noncases.

**Table 3:** Risk Reclassification Table of 10‐Year Major Osteoporotic Fracture (MOF) and Hip Fracture Stratified by Event Status^a^

Outcome	Model containing only FRAX score	Model containing FRAX score and additional variables^b^		
Hip fracture	Frequency (row %)	<3% risk	≥3% risk	Total
	Participants with an event			
	<3% risk	947 (72.4)	361 (27.6)	1308
	≥3% risk	91 (13.6)	579 (86.4)	670
	Total	1038	940	1978
	Participants without an event			
	<3% risk	76,119 (91.2)	7381 (8.8)	83,500
	≥3% risk	1906 (28.9)	4691 (71.1)	6597
	Total	78025	12072	90,097
Major osteoporotic fracture	Frequency (row %)	<20% risk	≥20% risk	Total
	Participants with a MOF event			
	<20% risk	8558 (89.9)	959 (10.1)	9517
	≥20% risk	291 (13.3)	1902 (86.7)	2193
	Total	8849	2861	11,710
	Participants without a MOF event			
	<20% risk	74,081 (96.4)	2747 (3.6)	76,828
	≥20% risk	1332 (22.7)	4543 (77.3)	5875
	Total	75,413	7290	82,703

a Net reclassification index (NRI) for cases:

Hip fracture: (361–91)/1978 = 0.137; MOF: (959–291)/11710 = 0.057.

NRI for noncases:

Hip: (1906–7381)/90097 = −0.061; MOF: (1332–2747)/82703 = −0.017.

b Treated diabetes, ≥two falls in the past year, vasomotor symptoms, physical function score.

All models are adjusted by Women**'**s Health Initiative Hormone and Calcium Vitamin D Trial intervention status (active, placebo, not randomized).

FRAX = Fracture risk assessment tool.

For hip fractures, there was a 13.7% improvement in reclassification among cases using the FRAX + additional risk factors model (all risk factors included simultaneously), compared with FRAX alone. Among women with hip fracture, 361 of 1978 participants who experienced hip fractures were correctly reclassified up (from low to high risk) with the FRAX + additional factors model compared with the FRAX model alone, and 91 of 1978 participants were incorrectly reclassified down (from high to low risk) with the FRAX + additional factors model. NRI values for noncases revealed no significant changes in reclassification for FRAX versus FRAX + additional risk factors.

In secondary analyses, we recalculated NRI separately for the FRAX model plus each of the additional risk factors individually compared with the FRAX model alone. The improvement in NRI by the model with FRAX + additional risk factors was likely driven by physical function (Supplementary Table SS1).

### Calibration of FRAX

**Figure 3:**
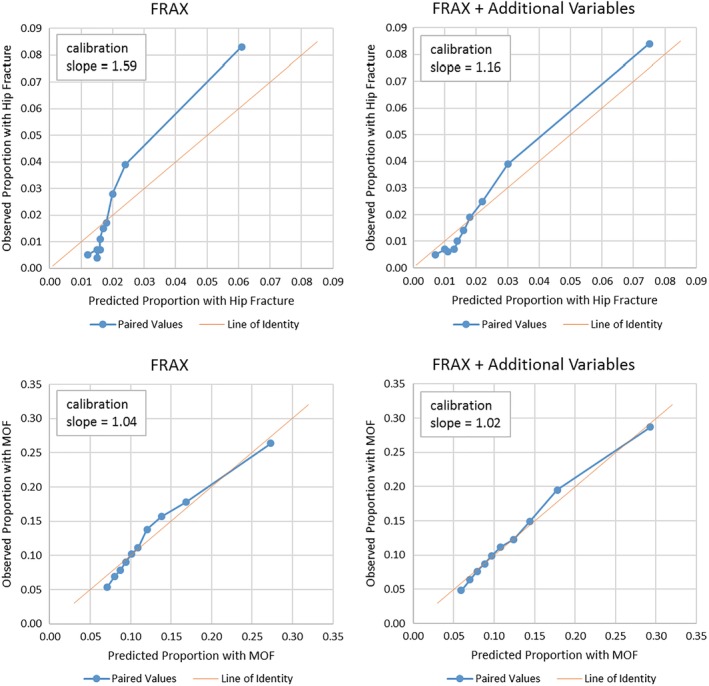
Observed and expected major osteoporotic fracture (MOF) events by decile of predicted fracture risk. Predicted fracture risk derived from a logistic regression model with fracture event, as a function of fracture risk assessment tool (FRAX) score alone, as well as with additional adjustments of treated diabetes, ≥two falls in the past year, vasomotor symptoms, and physical function score. All models are additionally adjusted for Women**'**s Health Initiative hormone and calcium vitamin D intervention assignments.

We assessed calibration (how closely the predicted absolute MOF probability matched the actual observed MOF probability) for FRAX without BMD information and for FRAX + additional risk factors both overall and for each decile of predicted MOF probability. The overall observed/predicted ratio was 1.00 for FRAX, as well as for FRAX + additional risk factors (Table 4). Within each decile of risk, calibration was good (approximately 1.0), with the exception of the lowest decile of predicted risk, where FRAX and FRAX + additional variables overestimated actual fracture probability (observed/predicted ratio 0.76 for FRAX, 0.81 for FRAX + additional risk factors). Figure 3 shows the predicted and observed MOF probabilities within each decile of predicted MOF risk.

**Table 4:** Observed and Expected Hip Fracture and MOF Events by Decile of Predicted Fracture Risk

		Hip fracture				Major osteoporotic fracture			
Model	Decile	*n*	Obs	Pred	Obs/Pred	*n*	Obs	Pred	Obs/Pred
FRAX	1	9218	0.005	0.012	0.42	9432	0.054	0.071	0.76
	2	9361	0.007	0.015	0.47	9452	0.069	0.080	0.86
	3	9047	0.004	0.015	0.27	9441	0.078	0.087	0.90
	4	9066	0.007	0.016	0.44	9432	0.090	0.094	0.96
	5	9267	0.011	0.016	0.69	9420	0.102	0.101	1.01
	6	9318	0.015	0.017	0.88	9468	0.111	0.109	1.02
	7	9172	0.017	0.018	0.94	9426	0.138	0.120	1.15
	8	9202	0.028	0.020	1.40	9467	0.157	0.138	1.14
	9	9223	0.039	0.024	1.63	9432	0.178	0.168	1.06
	10	9201	0.083	0.061	1.36	9441	0.264	0.273	0.97
	Total	92,075	0.021	0.021	1.00	94,413	0.124	0.124	1.00

Model	Decile	*n*	Obs	Pred	Obs/Pred	*n*	Obs	Pred	Obs/Pred
FRAX + additional variables^a^	1	9206	0.005	0.007	0.71	9440	0.048	0.059	0.81
	2	9210	0.007	0.010	0.70	9442	0.064	0.070	0.91
	3	9198	0.006	0.011	0.55	9440	0.076	0.079	0.96
	4	9215	0.007	0.013	0.54	9443	0.087	0.088	0.99
	5	9208	0.010	0.014	0.71	9441	0.099	0.097	1.02
	6	9208	0.014	0.016	0.88	9442	0.112	0.108	1.04
	7	9208	0.019	0.018	1.06	9441	0.123	0.124	0.99
	8	9208	0.025	0.022	1.14	9442	0.149	0.144	1.03
	9	9207	0.039	0.030	1.30	9442	0.195	0.178	1.10
	10	9207	0.084	0.075	1.12	9440	0.287	0.293	0.98
	Total	92,075	0.021	0.021	1.00	94,413	0.124	0.124	1.00

All models are adjusted by Women**'**s Health Initiative Hormone and Calcium Vitamin D Trial intervention status (active, placebo, not randomized).

Obs = Observed proportion; Pred = predicted proportion; FRAX = fracture risk assessment tool.

a Treated diabetes, ≥two falls in the past year, vasomotor symptoms, and physical function score.

FRAX was less well‐calibrated for hip fractures than for a MOF. At lower deciles of predicted risk, observed/predicted ratios were 0.27 to 0.47, indicating overestimation of risk by FRAX, whereas at the higher deciles of predicted risk, ratios were 1.4 to 1.6, indicating underestimation of risk by FRAX. This is reflected in the calibration slope for FRAX prediction of hip fractures, which was 1.59.

### Sensitivity analysis

When we included women who contributed less than 10 years of follow‐up (ie, dropped out of follow‐up or died), the AUC for all of the models were slightly lower. For example, AUC was 0.64 for the FRAX model without BMD information and 0.69 for the FRAX model with BMD information (Supplementary Table S2).

When we limited the analysis to participants who were not taking menopausal hormone therapy (Supplementary Table S3), AUC values were very similar to those of the primary analysis, and AUC values were not improved in the model containing FRAX + all additional factors compared with the FRAX model (Table 2). Among women aged 65 years or older at baseline (Supplementary Table S4 **)**, AUC values were lower (discrimination was lower) than among the analytic sample as a whole, but the main findings were consistent with those of the primary analysis.

## Discussion

In this large prospective study of postmenopausal women, the addition of diabetes, frequent falls, vasomotor symptoms, physical function, and lumbar spine BMD did not substantially improve reclassification of women with and without a MOF. We found a 6% improvement in correctly classifying among MOF cases, which is of questionable clinical relevance, and no evidence of a change in classification among controls. FRAX (with or without BMD information) had suboptimal performance in discriminating between women who did and did not experience MOF during the 10‐year study follow‐up period. As has been reported previously, FRAX had good performance in discriminating between women who did and did not experience a hip fracture during the follow‐up period. Of note, for both hip fractures and MOFs, AUC values for FRAX without BMD information (0.65 for MOF) and FRAX with BMD information (0.70 for MOF) were no better than simple models (age alone or age + BMI) and were not improved by the addition of other osteoporosis risk factors, including diabetes, frequent falls, vasomotor symptoms, physical function, and lumbar spine BMD in prediction of either fracture outcome. For MOFs and for hip fractures, discrimination between women with and without fractures was lower among women aged 65 years and older than in the overall study population.

Although discrimination of FRAX for MOF prediction was poor in this study, the calibration of FRAX for predicting a MOF was good. In contrast, we found that FRAX overestimated actual hip fracture risk among women at lower estimated risk, and underestimated hip fracture risk among women at higher estimated risk. Calibration and discrimination are distinct concepts; a given tool can have excellent calibration while simultaneously having suboptimal discrimination. Calibration is a measure of how well expected (predicted) events correspond with the actual observed events. Specifically, calibration describes whether the FRAX‐predicted fracture probabilities matched the actual observed cumulative fracture probabilities in a population or selected subgroups. In contrast, discrimination is a measure of how well a tool distinguishes between persons who do and do not have an event. In this study, discrimination refers to how well the FRAX model discriminated between individual persons who did and did not have an incident fracture outcome.

The improvement in correct classification of women who had a MOF by the model with FRAX + additional risk factors (treated diabetes, frequency of falls, vasomotor symptoms, and physical function score, simultaneously) compared with FRAX alone was 6%. The improvement in classification did not come with a loss of specificity. The improvement in correct classification of women who had hip fractures by the model with FRAX + additional risk factors was 14%. For both hip fractures and MOF, of the individual risk factors added to FRAX, this improvement was likely driven by the addition of physical function to FRAX. There is no widely accepted universal cutoff value that represents the minimum reclassification that is “clinically significant.”^33^ The interpretation of the clinical relevance of the reclassification results is somewhat subjective. However, we believe that 6% does not represent a notably clinically significant improvement, whereas 14% (for the FRAX model + additional risk factors in prediction of hip fractures) would qualify as clinically significant. We used clinically relevant risk categories (thresholds of 3% for hip fracture and 20% for MOF)^34^ in the reclassification analysis. The AUC values obtained using FRAX (alone or with additional risk factors) for prediction of a MOF in this study, were in the range of 0.64 to 0.67; AUC values less than 0.7 are generally considered to be not clinically useful.

Our results have clinical importance. We focused on examining the role of common clinical risk factors that are not included in the FRAX model. Type 2 diabetes is increasingly diagnosed in the United States. Eleven percent of women in the United States have diabetes, and the incidence of diabetes increase with age.^35^ Falls and impaired physical function will become increasingly more common with the aging of the US population. In 2014, 29% of older adults in the United States reported an estimated 29 million falls in the preceding 12 months.^36^ Frequent vasomotor symptoms last more than 7 years for more than half of women^37^ and for 10 years or more in more than one‐third of women.^38^ We previously found vasomotor symptoms to be associated with increased fracture risk among WHI participants.^16^ However, none of these common risk factors enhanced the ability of FRAX to discriminate between women who did and did not experience a MOF.

Among women, lumbar spine osteoporosis is more prevalent than femoral neck osteoporosis, highlighting the potential importance of considering the lumbar spine BMD in fracture risk assessment tools. Menopause‐related losses in BMD are disproportionately higher at the lumbar spine than at the hip.^39^ About 10% of US women ≥50 years old have lumbar spine BMD in the osteoporotic range.^13^ In the Study of Osteoporotic Fractures (white women aged ≥65 years), 16% were osteoporotic at the lumbar spine, but not at the hip.^14^ In such a situation, FRAX would be expected to underestimate fracture risk because it does not include lumbar spine BMD values in risk prediction. A retrospective cross‐sectional study suggested that for women younger than 60 years, the odds of having a fracture based on the presence of lumbar spine osteoporosis was greater than that based on femoral neck osteoporosis.^40^ Although lumbar spine BMD information did not enhance the ability of FRAX to discriminate between women who did and did not experience MOF in our current study, a procedure based on the difference (offset) between lumbar spine and femoral neck *T*‐scores may enhance fracture prediction by FRAX.^11^, ^12^ Nonetheless, in our study, the discrimination of FRAX for prediction of MOF was not improved by consideration of those additional risk factors in addition to FRAX.

FRAX is designed in part to account for competing mortality risk as it incorporates country‐specific death rates into its calculation. However, discrimination of MOF was if anything slightly lower in models that included women who did not survive the entire 10‐year follow‐up period. These findings suggest that fracture risk assessment tools that provide individual‐based estimates of long‐term fracture probability might be improved by incorporation of individual patient‐based estimates of competing mortality risk.

Our results were similar to those of a study of Chinese women aged ≥65 years; that study showed no improvement in reclassification comparing FRAX + recurrent falls to FRAX alone for a MOF.^41^ In elderly men, the AUC for a model containing FRAX with BMD information + falls was 0.61, which was no better than a high FRAX score (predicted MOF risk ≥20%) alone (AUC 0.72) for predicting a MOF.^42^ We are not aware of other studies evaluating the other variables that we examined in this study (diabetes, vasomotor symptoms, lumbar spine BMD, and physical function) in relation to improving the prediction of a MOF by FRAX.

The discrimination of FRAX for predicting a MOF was lower in black women than in white women. We suspect that this finding is because of the lower precision (and lower incidence rates) of MOFs in black women than in white women.

Although efforts to improve discrimination of the FRAX model for MOFs have yielded disappointing results, there may be additional (as yet unidentified) risk factors that are available in administrative and electronic health record systems that may be productive avenues of inquiry in future efforts to improve the discrimination of the FRAX tool and other fracture prediction tools, such as QFracture.

Limitations of this study include lack of information about other potential risk factors, including bone microarchitecture (eg, cortical porosity), trabecular bone score, hip axis length, vertebral imaging, bone turnover marker levels, and objective measures of physical function. In previous studies, two DXA‐derived measures (hip axis length^43^ and the trabecular bone score adjustment to FRAX^44^, ^45^) each provided improvement in net reclassification compared with the FRAX model alone. A study of Japanese women aged ≥40 years showed improvement in risk reclassification for the FRAX with trabecular bone score compared with the FRAX model alone.^46^ A study of Swedish women aged 69 to 79 years showed improvement in risk reclassification with the addition of gait speed and one‐leg standing time.^47^ In addition, except for hip fractures, fractures were self‐reported in this study. However, a prior study of the WHI showed that agreement between self‐report and medical record review was 71%.^48^ Finally, because the FRAX model coefficients are proprietary, we could not account for the specific risk factor weighting of the risk factors in the FRAX model.

This study had several strengths, including prospective assessment of incident fractures for 10 years, detailed information regarding osteoporosis risk factors, and large sample size.

In conclusion, in this cohort of community‐dwelling US postmenopausal women, the performance of FRAX + additional selected risk factors in predicting risk of a MOF was similar to that based on FRAX alone.

## Disclosures

The following authors declare that they have no conflicts of interest: CJC, JAR, JTS, KEE, JL, JAC, AZL. NBW is a speaker for Amgen and Radius and a consultant for Radius.

## Supporting information


**Supplementary Table S1.** Risk Reclassification Table of 10‐Year Major Osteoporotic Fracture and Hip Fracture, Stratified by Individual Variables Included in the Model*
**Supplementary Table S2.** AUC values for Fracture Risk Assessment Tool (FRAX) alone and FRAX with additional clinical characteristics on 10‐year major osteoporotic fracture, including participants with less than 10 years of follow‐up (regardless of fracture status)
**Supplementary Table S3.** Area under the Receiver Operating Characteristic Curve Values for Fracture Risk Assessment Tool (FRAX) Alone and FRAX with Additional Clinical Characteristics on Predicted 10‐year Risk of Fracture Among Women Not Taking Hormone Therapy*
**Supplementary Table S4.** Area under the Receiver Operating Characteristic Curve Values for Fracture Risk Assessment Tool (FRAX) Alone and FRAX with Additional Clinical Characteristics on Predicted 10‐year Risk of Fracture Among Women Aged ≥65 Years.
