# Supplementary material for: Do Additional Clinical Risk Factors Improve the Performance of Fracture Risk Assessment Tool (FRAX) Among Postmenopausal Women? Findings From the Women's Health Initiative Observational Study and Clinical Trials
Source: JBMR Plus. 2019 Nov 30;3(12):e10239. doi: 10.1002/jbm4.10239 (PMC6894725; doi:10.1002/jbm4.10239)
Supplement: Supplementary file 1 — Supplementary Table S1. Risk Reclassification Table of 10‐Year Major Osteoporotic Fracture and Hip Fracture, Stratified by Individual Variables Included in the Model* Supplementary Table S2. AUC values for Fracture Risk Assessment Tool (FRAX) alone and FRAX with additional clinical characteristics on 10‐year major osteoporotic fracture, including participants with less than 10 years of follow‐up (regardless of fracture status) Supplementary Table S3. Area under the Receiver Operating Characteristic Curve Values for Fracture Risk Assessment Tool (FRAX) Alone and FRAX with Additional Clinical Characteristics on Predicted 10‐year Risk of Fracture Among Women Not Taking Hormone Therapy* Supplementary Table S4. Area under the Receiver Operating Characteristic Curve Values for Fracture Risk Assessment Tool (FRAX) Alone and FRAX with Additional Clinical Characteristics on Predicted 10‐year Risk of Fracture Among Women Aged ≥65 Years. [file JBM4-3-na-s001.pdf]

Supplemental Tables

Do additional clinical risk factors improve the performance of Fracture Risk Assessment Tool (FRAX) Among Postmenopausal Women? Findings from the Women's Health Initiative Observational Study and Clinical Trials

Carolyn J. Crandall, MD, MS, Joseph Larson, MS, Jane A. Cauley, DrPH, John T. Schousboe, MD, PhD, Andrea Z. LaCroix, PhD, John A. Robbins, MD, Nelson B. Watts, MD, Kristine E. Ensrud, MD, MPH

**Table**	**Page #**
Supplemental Table 1. Risk Reclassification Table of 10-Year Major Osteoporotic Fracture and Hip Fracture, Stratified by Individual Variables Included in the Model	2
Supplemental Table 2. AUC values for Fracture Risk Assessment Tool (FRAX) alone and FRAX with additional clinical characteristics on 10-year major osteoporotic fracture, including participants with less than 10 years of follow-up (regardless of fracture status)	3
Supplemental Table 3. Area under the Receiver Operating Characteristic Curve Values for Fracture Risk Assessment Tool (FRAX) Alone and FRAX with Additional Clinical Characteristics on Predicted 10-year Risk of Fracture Among Women Not Taking Hormone Therapy	4
Supplemental Table 4. Area under the Receiver Operating Characteristic Curve Values for Fracture Risk Assessment Tool (FRAX) Alone and FRAX with Additional Clinical Characteristics on Predicted 10-year Risk of Fracture Among Women Aged ≥65 Years	5

Supplemental Table 1. Risk Reclassification Table of 10-Year Major Osteoporotic Fracture and Hip Fracture, Stratified by Individual Variables Included in the Model[*]

Outcome	Variable added to model containing FRAX	Outcome Type	NRI[1]
Hip Fracture (High risk ≥3%)	Diabetes	Case	0.04
		Non-Case	-0.02
	Falls	Case	0.02
		Non-Case	-0.01
	Vasomotor Symptoms	Case	0.02
		Non-Case	-0.01
	Physical Function	Case	0.11
		Non-Case	-0.05
	All Additional Factors	Case	0.14
		Non-Case	-0.06
Osteoporotic Fracture (High risk ≥20%)	Diabetes	Case	0.01
		Non-Case	-0.00
	Falls	Case	0.01
		Non-Case	-0.00
	Vasomotor Symptoms	Case	0.00
		Non-Case	-0.00
	Physical Function	Case	0.04
		Non-Case	-0.01
	All Additional Factors	Case	0.06
		Non-Case	-0.02

[*] For cases, NRI calculates and tests being reclassified into the high-risk category; For non-cases, NRI calculates and tests being reclassified into the low-risk category.

Supplemental Table 2. AUC values for Fracture Risk Assessment Tool (FRAX) alone and FRAX with additional clinical characteristics on 10-year major osteoporotic fracture, including participants with less than 10 years of follow-up (regardless of fracture status)

Model[*]	n	AUC (95% CI)
**All Participants**		
Age	129439	61.9 (61.4, 62.4)
Age + body mass index	129439	62.0 (61.5, 62.5)
Age + history of fracture (any site)	129439	62.7 (62.1, 63.2)
FRAX alone (all participants)	129439	64.2 (63.6, 64.7)
FRAX alone (white participants)	107906	62.2 (61.6, 62.7)
FRAX alone (black participants)	10960	57.5 (54.3, 60.7)
FRAX + treated diabetes	129439	64.2 (63.7, 64.7)
FRAX + ≥2 falls in the past year	129439	64.5 (63.9, 65.0)
FRAX + vasomotor symptoms	129439	64.0 (63.6, 64.6)
FRAX + physical function	129439	64.5 (63.9, 65.0)
FRAX + all additional factors	129439	64.7 (64.2, 65.3)
**BMD Subset**		
Age	8247	62.2 (60.1, 64.2)
Age + body mass index	8247	62.2 (60.2, 64.2)
Age + history of fracture (any site)	8247	63.9 (61.9, 66.0)
FRAX alone (all participants)	8247	69.2 (67.3, 71.1)
FRAX alone (white participants)	6410	66.2 (64.1, 68.3)
FRAX alone (black participants)	1222	65.2 (54.6, 75.9)
FRAX + treated diabetes	8247	69.3 (67.4, 71.2)
FRAX + ≥2 falls in the past year	8247	69.3 (67.3, 71.2)
FRAX + vasomotor symptoms	8247	69.1 (67.2, 71.0)
FRAX + physical function	8247	69.2 (67.3, 71.2)
FRAX + lumbar spine bone mineral density	8247	69.1 (67.2, 71.0)
FRAX + all additional factors	8247	69.5 (67.5, 71.4)

[*] All models are adjusted for intervention assignment in the WHI Hormone (Active, Placebo, not Randomized) and Calcium Vitamin D (Active, Placebo, Not Randomized) trials. BMD: bone mineral density.

Supplemental Table 3. Area under the Receiver Operating Characteristic Curve Values for Fracture Risk Assessment Tool (FRAX) Alone and FRAX with Additional Clinical Characteristics on Predicted 10-year Risk of Fracture Among Women Not Taking Hormone Therapy*

	**Hip Fracture**		**Major Osteoporotic Fracture**	
**Model**	n	AUC (95% CI)	n	AUC (95% CI)
All Participants				
Age	45431	76.6 (75.2, 77.9)	46980	64.1 (63.4, 64.8)
Age + BMI	45431	77.0 (75.7, 78.3)	46980	64.2 (63.5, 64.9)
Age + hx fracture (any site)	45431	76.6 (75.2, 77.9)	46980	64.6 (63.9, 65.4)
FRAX alone (all participants)	*45431*	*76.6 (75.3, 77.9)*	*46980*	*65.0 (64.3, 65.7)*
FRAX alone (white participants)	38551	75.4 (74.0, 76.8)	39897	63.6 (62.9, 64.4)
FRAX alone (black participants)	3969	79.9 (71.3, 88.5)	4044	60.2 (56.3, 64.1)
FRAX + treated diabetes	45431	76.2 (74.9, 77.5)	46980	65.2 (64.5, 65.9)
FRAX + ≥2 falls in the past year	45431	75.5 (74.1, 76.9)	46980	65.3 (64.6, 66.0)
FRAX + vasomotor symptoms	45431	74.7 (73.2, 76.2)	46980	65.0 (64.3, 65.7)
FRAX + physical function	45431	75.2 (73.8, 76.6)	46980	66.0 (65.3, 66.7)
FRAX + all additional factors	45431	75.2 (73.7, 76.6)	46980	66.4 (65.7, 67.1)
**BMD Subset**				
Age	2917	73.3 (68.2, 78.4)	3044	65.8 (63.2, 68.4)
Age + BMI	2917	73.6 (68.4, 78.7)	3044	65.7 (63.2, 68.3)
Age + hx fracture (any site)	2917	73.4 (68.4, 78.4)	3044	67.2 (64.6, 69.8)
FRAX alone (all participants)	*2917*	*80.2 (75.9, 84.5)*	*3044*	*71.2 (68.8, 73.7)*
FRAX alone (white participants)	2331	77.9 (73.2, 82.7)	2440	68.4 (65.6, 71.1)
FRAX alone (black participants)	407	79.9 (61.1, 98.7)	413	69.4 (56.1, 82.6)
FRAX + treated diabetes	2917	80.0 (75.6, 84.4)	3044	71.4 (68.9, 73.8)
FRAX + ≥2 falls in the past year	2917	78.7 (74.2, 83.3)	3044	71.6 (69.1, 74.0)
FRAX + vasomotor symptoms	2917	80.0 (75.0, 84.1)	3044	71.3 (68.9, 73.8)
FRAX + physical function	2917	78.7 (74.3, 83.0)	3044	72.6 (70.2, 75.0)
FRAX + lumbar spine BMD	2917	78.5 (74.0, 83.0)	3044	71.0 (68.6, 73.5)
FRAX + all additional factors	2917	80.0 (75.6, 84.4)	3044	73.2 (70.8, 75.6)

* BMD: Bone mineral density

Supplemental Table 4. Area under the Receiver Operating Characteristic Curve Values for Fracture Risk Assessment Tool (FRAX) Alone and FRAX with Additional Clinical Characteristics on Predicted 10-year Risk of Fracture Among Women Aged ≥65 Years

	Hip Fracture		Osteoporotic Fracture	
**Model[1]**	n	AUC (95% CI)	n	AUC (95% CI)
**All Participants**				
Age	38227	69.4 (68.1, 70.8)	39771	60.3 (59.6, 61.0)
Age + BMI	38227	70.0 (68.6, 71.3)	39771	60.3 (59.6, 61.0)
Age + hx fracture (any site)	38227	69.6 (68.2, 71.0)	39771	61.3 (60.6, 62.1)
FRAX alone (all participants)	*38227*	*69.0 (67.7, 70.4)*	*39771*	*61.3 (60.6, 62.0)*
FRAX alone (white participants)	34375	68.5 (67.1, 69.9)	35765	60.5 (60.0, 61.3)
FRAX alone (black participants)	1860	77.2 (69.9, 84.5)	1906	62.5 (58.0, 67.1)
FRAX + treated diabetes	38227	69.7 (68.3, 71.0)	39771	61.7 (61.0, 62.5)
FRAX + ≥2 falls in the past year	38227	68.7 (67.3, 70.0)	39771	61.9 (61.2, 62.6)
FRAX + vasomotor symptoms	38227	68.7 (67.3, 70.0)	39771	61.3 (60.6, 62.0)
FRAX + physical function	38227	69.5 (68.2, 70.9)	39771	62.9 (62.2, 63.7)
FRAX + all additional factors	38227	70.0 (68.7, 71.4)	39771	63.5 (62.8, 64.2)
**BMD Subset**				
Age	2363	67.7 (62.5, 73.0)	2498	61.8 (59.0, 64.6)
Age + BMI	2363	68.3 (63.1, 73.5)	2498	61.8 (59.0, 64.7)
Age + hx fracture (any site)	2363	67.7 (62.4, 72.9)	2498	63.7 (60.9, 66.5)
FRAX alone (all participants)	*2363*	*72.0 (67.0, 77.0)*	*2498*	*67.7 (65.1, 70.3)*
FRAX alone (white participants)	2067	71.7 (66.5, 76.9)	2190	66.4 (63.7, 69.2)
FRAX alone (black participants)	198	81.2 (75.8, 86.7)	202	75.0 (62.4, 87.6)
FRAX + treated diabetes	2363	73.1 (68.2, 78.1)	2498	67.9 (65.3, 70.5)
FRAX + ≥2 falls in the past year	2363	73.1 (68.1, 78.0)	2498	68.1 (65.5, 70.7)
FRAX + vasomotor symptoms	2363	71.8 (66.8, 76.8)	2498	67.7 (65.1, 70.3)
FRAX + physical function	2363	72.0 (67.0, 77.0)	2498	69.2 (66.6, 71.8)
FRAX + lumbar spine BMD	2363	71.7 (66.7, 76.6)	2498	67.4 (64.8, 70.0)
FRAX + all additional factors	2363	75.3 (70.6, 80.1)	2498	70.0 (67.4, 72.6)
